# Supplementary material for: The Healthcare and Societal Costs of Familial Intellectual Disability
Source: Int J Environ Res Public Health. 2024 Mar 4;21(3):299. doi: 10.3390/ijerph21030299 (PMC10970490; doi:10.3390/ijerph21030299)
Supplement: Supplementary file 1 [file ijerph-21-00299-s001.zip › Supplementary Table S1.pdf]

Supplementary Table S1: Source of Cost Estimates and Attributing Perspective of Items Surveyed[1]

Item	Source of Cost Estimates	Perspective
Utilisation of Healthcare Services – ID Participant		
Hospital Admissions	AR-DRG and linked health data	Government
Emergency Department Admissions	URG and linked data	Government
Medical Services (general practitioner, neurologist, paediatrician, psychiatrist, other)	Linked Medicare data	Government and Private OOP
Allied Health (physiotherapy, occupational therapy, speech pathology, psychology/counselling/ social work, podiatrist, dentist, nutritionist/dietician, optometrist, other)	Linked Medicare data	Government and Private OOP
Prescription Medication	Linked Medicare data	Government and Private OOP
Complimentary health (naturopath, chiropractor, osteopath, other)	Survey responses	Private OOP
Alternative medicines (herbal medicines, natural remedies, special diet supplements, other)	Survey responses	Private OOP
Non-prescriptions Medication	Survey responses	Private OOP
Accommodation and Respite Care		
ID Participant Main Place of Residence (home, residential care, specialised disability accommodation (SDA), supported independent living (SIL), public housing, with another relative, in a separate house owned by carer)	Residential care fees, including the accommodation fee and the basic daily living fee[2]	Government
	SIL Accommodation fee[2]	Government
	Public Housing Rent: Up to 25-30% less than private market rent for tenants[2]	Government and Private OOP
Use of Respite Care (in-home, out-of-home, residential respite care)	Respite Care Fees[2]	Government
Government Transfer Payments		
Welfare payments (no welfare payments, age pension, Newstart allowance, parenting payment or parenting payment single, service pension, disability support pension (paid by Centrelink or Department of Veteran Affairs), wife pension or widow allowance, partner allowance,	Survey Responses	Government

sickness allowance or special benefit, youth allowance (as a full-time student or jobseeker), Austudy/Abstudy, mobility allowance, rent assistance, carer payment and carer allowance, other)		
Education Support		
Previous 12 months public or private education attendance at: early intervention centre, preschool/childcare, school mainstream class with aide, school mainstream class without an aide, school mild ID class, school other ID class, school only for children with disabilities, technical and further education (TAFE)/technical school, residential treatment centre or school	Survey Response and government school fees contribution depending on level of support[2]	Government and Private OOP
Utilisation of Nappies, Aids, Appliances and Modifications, and Transport Costs		
Nappies	Survey Responses	Government and Private OOP
Aids and appliances (walking aids/crutches, standard wheelchair, electric wheelchair, lifting apparatus, peg feeding equipment, visual aids (includes glasses, braille books), hearing aids, communication aids, orthotics/special shoes, other)	Survey Responses	Government and Private OOP
Modifications to care or home (stairs, bedroom, external, bathrooms, hallways, kitchen, living room, other)	Survey Responses	Government and Private OOP
Transport cost and subsidy (none, bus service, taxi transport subsidy scheme, other)	Survey Responses	Government and Private OOP
Income and Employment		
Previous 12 months debt, savings and income (earned income, interest from savings, welfare payments, other)	Survey Responses	Government and Private OOP
Carer and spouse work status (full-time, including maternity leave or other paid leave, part-time (includes maternity or other paid leave, unemployed and looking for work, not employed and not looking for work)	Survey Responses	Government and Private OOP
ID participant employment in previous 12 months (open/unsupported employment,	Survey Responses	Government and Private OOP

supported employment, Australian Disability Enterprise (Sheltered Workshop), family employment, adult day services, TAFE/technical school (supported access scheme, transition to work scheme)		

1.To year 2021

2.For further details see Schofield, D. et al (2022) [20]
