# Supplementary material for: The Healthcare and Societal Costs of Familial Intellectual Disability
Source: Int J Environ Res Public Health. 2024 Mar 4;21(3):299. doi: 10.3390/ijerph21030299 (PMC10970490; doi:10.3390/ijerph21030299)
Supplement: Supplementary file 1 [file ijerph-21-00299-s001.zip › Supplementary Table S2.pdf]

Supplementary Table S2. Estimated average annual cost (standard error) by age group, 2021 AUD

		ID Participant Average Annual Cost									Carer and Spouse Average Annual Cost						
		Commonwealth Government			State Government		Private OOP				Commonwealth Government		State Government	Private OOP			
Age of ID Participant	*n*	Healthcare	Societal[1]	Taxes lost	Healthcare	Societal[2]	Healthcare	Societal[3]	Lost income	ID Participant Total Average Annual Cost[4]	Societal[5]	Taxes lost	Societal	Carer and spouse lost income	Carer and Spouse Total Average Annual Cost[4]	Total Annual Average Cost per Person	Total Annual Average Cost per Household
0 to 4	9	$12,381 ($5,984)	$899 ($842)	$0 ($0)	$8,664 ($6,325)	$0 ($0)	$624 ($236)	$13,326 ($9,562)	$0 ($0)	$35,894 ($16,130)	$10,775 ($4,368)	$7,190 ($8,284)	$0 ($0)	$15,386 ($6,346)	$15,387 ($6,346)	$51,281 ($18,785)	$79,608 ($29,161)
5 to 18	85	$8,037 ($990)	$31,076 ($5,456)	$627 ($142)	$2,751 ($772)	$20,547 ($1,623)	$1,034 ($362)	$2,131 ($554)	$ 5,134 ($998)	$69,574 ($5,973)	$9,004 ($1,006)	$8,556 ($1,545)	$998 ($432)	$10,296 ($1,806)	$11,293 ($1,989)	$80,867 ($6,222)	$125,536 ($9,659)
19 to 29	35	$3,489 ($693)	$59,410 ($11,498)	$14,076 ($1,236)	$624 ($200)	$0 ($0)	$358 ($125)	$1,060 ($249)	$44,605 ($ 3,161)	$87,654 ($11,616)	$7,098 ($1,488)	$3,600 ($1,530)	$1,960 ($997)	$8,352 ($2,167)	$10,311 ($2,249)	$97,965 ($11,255)	$152,080 ($11,472)
30 to 39	15	$3,523 ($547)	$114,816 ($34,348)	$28,647 ($1,229)	$1,284 ($412)	$724 ($724)	$227 ($100)	$377 ($218)	$ 91,248 ($ 3,823)	$189,783 ($33,573)	$4,399 ($1,547)	$1,836 ($1,583)	$1,850 ($1,261)	$6,594 ($2,064)	$8,445 ($2,809)	$198,228 ($33,833)	$307,725 ($52,522)
40 to 60	19	$8,782 ($4,038)	$96,858 ($20,620)	$9,638 ($1,926)	$5,635 ($3,999)	$2,285 ($1043)	$738 ($218)	$1,295 ($1,098)	$ 31,482 ($ 8,783)	$119,489 ($27,178)	$6,087 ($1,806)	$380 ($2,854)	$0 ($0)	$0 ($0)	$0 ($0)	$119,489 ($27,178)	$185,492 ($42,191)

Notes:
[1]Comprises of welfare payments, special education and disability supports (including NDIS support accommodation, aids, appliances, and modifications to house or car costs.
[2]Comprises of accommodation supports and special education costs.
[3]Expenses for aids, home and vehicle modification, accommodation, supplements/non-prescription medicines/special diets.
[4]Welfare payments and taxes lost were excluded to avoid double counting.
[5]Comprises of welfare payments and housing support costs.
